# Predictive and Prognostic Utility of the Serum Level of Resistin-Like Molecule Beta for Risk Stratification in Patients with Community-Acquired Pneumonia

**DOI:** 10.3390/pathogens10020122

**Published:** 2021-01-25

**Authors:** Li Chen, Qiongzhen Luo, Ying Shang, Xinwei He, Yu Xu, Zhancheng Gao

**Affiliations:** 1 Department of Respiratory & Critical Care Medicine, Peking University People’s Hospital, Beijing 100044, China; 1811110324@bjmu.edu.cn (L.C.); lqza02677@btch.edu.cn (Q.L.); heyukun@bjmu.edu.cn (Y.S.); 2 Department of Internal Medicine, Xicheng District Zhanlanlu Hospital, Beijing 100032, China; 1911110323@pku.edu.cn

**Keywords:** community-acquired pneumonia, severity, mortality, resistin-like molecule beta

## Abstract

Despite progress in intensive care, the morbidity and mortality of patients with community-acquired pneumonia (CAP) remains high. Furthermore, the predictive and prognostic utility of resistin-like molecule beta (RELM-β) in patients with CAP is uncertain. This study investigated the role of RELM-β in patients with CAP and evaluated its correlation with disease severity and the risk of death. A prospective, multicenter study was conducted in 2017, and admission serum levels of RELM-β were detected using quantitative enzyme-linked immunosorbent assay. A total of 114 and 112 patients with severe CAP (SCAP) and non-severe CAP (NSCAP) were enrolled, respectively, with 15 healthy controls. Patients with SCAP, especially non-survivors, had significantly higher levels of serum RELM-β than patients with NSCAP. RELM-β levels positively correlated with severity scores and consistently predicted SCAP in patients with CAP (area under the curve = 0.794). Increased levels of RELM-β were closely related to the severity and prognosis of patients with CAP. The accuracy of 30-day mortality predictions of CURB-65 (confusion, urea, respiratory rate, blood pressure, and age ≥ 65 years) can be significantly improved when combined with RELM-β levels. The level of RELM-β can assist clinicians in risk stratification of patients with CAP in early stages.

## 1. Introduction

Community-acquired pneumonia (CAP) is an infectious disease found worldwide [1]. It has high rates of morbidity and mortality and exacts high economic costs [2]. Despite recent advancements in antimicrobial therapy, the CAP mortality rate remains high (18% in hospitalized patients) and can exceed 50% in patients with severe CAP (SCAP) [3]. Higher mortality rates are correlated with the inadequate treatment of outpatients and the delayed admission of patients with CAP [4]. The most challenging tasks for a physician is the identification of risks for patients with CAP and the subsequent administration of individualized treatment [5]. Moreover, a significant number of patients with pneumonia who eventually developed SCAP and were transferred to the intensive care unit did not show obvious severe symptoms on initial admission [5,6]. Several predictors of prognosis and mortality in individuals with CAP have been developed to identify individuals who are at risk of poor outcomes as early as possible [7,8]. The most widely recommended scoring methods are the pneumonia severity index (PSI) and CURB-65 (confusion, urea, respiratory rate, blood pressure, and age ≥ 65 years), both of which have been widely vetted [9]. However, the PSI score depends on preexisting conditions and age, and CURB-65 does not accurately predict mortality and poorly assesses CAP symptoms. Neither method evaluates the host inflammatory response, which is considered a critical aspect of CAP prognosis [10]. Biomarkers can identify risks simply, objectively, and effectively; therefore, they are helpful for clinical assessment [11,12].

Resistin-like molecules (RELMs) are small, secreted proteins and their family members (RELM-α, RELM-β, resistin, and RELM-γ) are found in the intestine, lungs, and adipose tissue [13]. RELMs are associated with certain inflammatory diseases and metabolic pathophysiology [13,14]. Prior research has shown RELM-β is a profibrotic mediator in diseases related to the airway and is regulated in immune-responses and respiratory infections [15]. RELM-β appears to belong to a family of mediators that may be downstream of the immune response and both amplify the inflammatory response and mediate some of its effects [16]. Moreover, RELM-β is an allergen-induced cytokine with inflammatory and remodeling activity in the murine lung [17]. Murine RELM-β was upregulated by ovalbumin challenge in the mouse acute pulmonary inflammation model [18]. There is currently a lack of research on the association between CAP prognosis and the severity and levels of serum RELM-β. In the current study, we hypothesized that higher RELM-β levels could indicate poor prognosis and mortality within 30 days. In addition, proadrenomedullin (proADM), a vasodilatory peptide with antimicrobial and anti-inflammatory properties, has shown promise in predicting illness severity in sepsis and lower respiratory tract infections in adults [19]. The level of proADM was also measured, and the predictive performance was evaluated and compared with that of RELM-β. Our goal was to clarify the precise role of RELM-β in CAP and assess the ability of this enzyme to predict the severity and risk of death in patients with CAP on admission. 

## 2. Results

### 2.1. Population Characteristics of Enrolled Patients 

The study population comprised 244 patients with a CAP diagnosis who met the criteria for inclusion and 15 healthy controls (HCs) as reference. Among these patients, 18 patients were later excluded because of hemolysis or insufficient clinical data. The final study population consisted of 226 patients (114 SCAP and 112 non-severe CAP (NSCAP) patients) with a median age of 57.50 years (interquartile range (IQR) 42.25–73.00). Nineteen of the patients with SCAP died within 30 days. Table 1 provides detailed information on the demographic and clinical characteristics of the 226 patients with CAP. The spectrum of infectious pathogens in patients with CAP is diverse, including bacteria, viruses, and atypical pathogens, which are typical for the heterogeneous CAP patient population. A comparison showed there were no significant differences in terms of age, sex, smoking history, maximum body temperature (T_max_), and antibiotic pretreatment between the SCAP and NSCAP groups (all *p* > 0.05). However, respiration frequencies and heart rates of patients with SCAP were higher than those of patients with NSCAP (all *p* < 0.05). Laboratory test analyses showed that indicators related to inflammatory responses, such as white blood cell (WBC), neutrophil percentage (NEU), neutrophil-to-lymphocyte ratio (NLR), C-reactive protein (CRP), erythrocyte sedimentation rate, and procalcitonin (PCT) of the SCAP group were significantly higher than those of the NSCAP group (all *p* < 0.05). Compared with that of the NSCAP group, the lymphocyte percentage (LYM) of the SCAP group was significantly lower (*p* < 0.0001). Among the SCAP group, the proportions of bilateral lung infection and pleural effusion were 84.21% and 32.46%, respectively, which were significantly higher than those of NSCAP patients (both *p* < 0.0001). The mortality rate of patients with SCAP was 16.67%, whereas all patients without SCAP improved or were discharged from the hospital within 30 days (Table 1).

### 2.2. Level of RELM-β in Each Group and Etiology 

**Figure 1:**
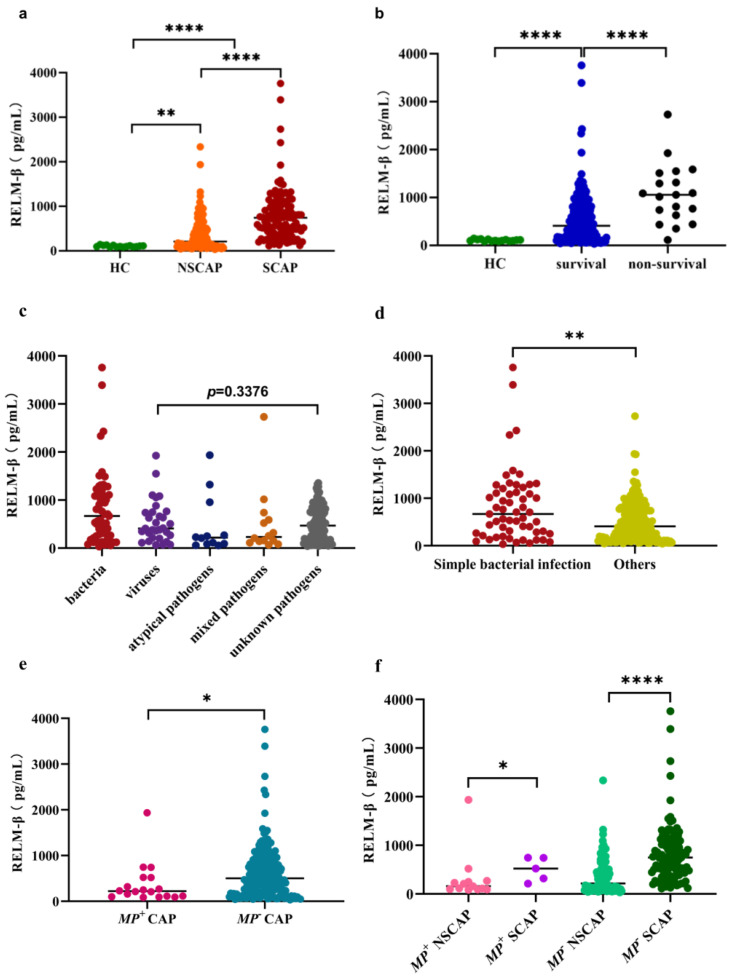
Levels of resistin-like molecule beta (RELM-β) in patients with community-acquired pneumonia (CAP) across multiple groups. (**a**) Levels of RELM-β in healthy controls (HCs), patients with non-severe CAP (NSCAP), and patients with severe CAP (SCAP). (**b**) Levels of RELM-β in healthy controls (HCs), survival and non-survival. (**c**) Comparison of RELM-β in patients with CAP infected with different pathogens. (**d**) Comparison of RELM-β in patients with CAP infected with one or more bacteria and others. The others include viral or atypical pathogen infections, mixed infections, or pathogens are unknown. (**e**) Levels of RELM-β in *Mycoplasma pneumonia*-positive (*Mp*^+^) patients with CAP and *Mycoplasma pneumonia*-negative (*Mp^−^*) patients with CAP. (**f**) Comparison of RELM-β in patients with *Mp*^+^ NSCAP, *Mp*^+^ SCAP, *Mp^–^* NSCAP and *Mp*^−^ SCAP. * *p* < 0.05, ** *p* < 0.01, *** *p* < 0.001, **** *p* < 0.0001.

Initially, we executed a cross-sectional comparison of RELM-β levels between CAP groups and an independent set of 15 prospectively recruited HCs. The serum RELM-β level in patients with CAP at admission was 468.70 (189.90–837.60) pg/mL, which was notably higher than that in the HC group (97.72 (89.57–121.60) pg/mL, *p* < 0.0001) (Figure 1a). Subgroup analysis showed that patients with SCAP exhibited higher levels of RELM-β than patients with NSCAP (740.70 (425.30–1063.00) and 209.00 (100.00–513.90), respectively; *p* < 0.0001). Furthermore, the serum RELM-β level in patients who eventually died within 30 days was 1074.00 ± 623.60 pg/mL, which was considerably higher than that in survivors (407.70 (168.90–798.80), *p* < 0.0001) (Figure 1b). 

According to the pathogen identified, the RELM-β levels of patients with bacterial infection, viral infection, atypical pathogen infection, mixed pathogen infection, and unknown pathogens were 668.40 (259.10–1164.0), 407.20 (213.30–747.70), 218.40 (85.41–782.70), 232.20 (134.60–626.00), and 468.70 (186.40–797.90) pg/mL, respectively (Figure 1c). Notably, the expression level of RELM-β in patients with CAP resulting from bacterial infection was significantly higher than that in patients infected with other pathogens (*p* = 0.0047) (Figure 1d). Eighteen (7.96%) patients were *Mycoplasma pneumonia (Mp)*-positive determined by serological testing. We found the RELM-β levels of patients in the *Mp*-positive group were significantly higher than those of *Mp*-negative patients (*p* = 0.022) (Figure 1e). Regardless of whether *Mp* was detected in the patients (*Mp*-positive group or *Mp*-negative group), the RELM-β levels of the SCAP group were significantly higher than those of the NSCAP group (*p* = 0.0264, *p* < 0.0001, respectively) (Figure 1f).

### 2.3. Correlation between Level of RELM-β and CAP Severity 

**Figure 2:**
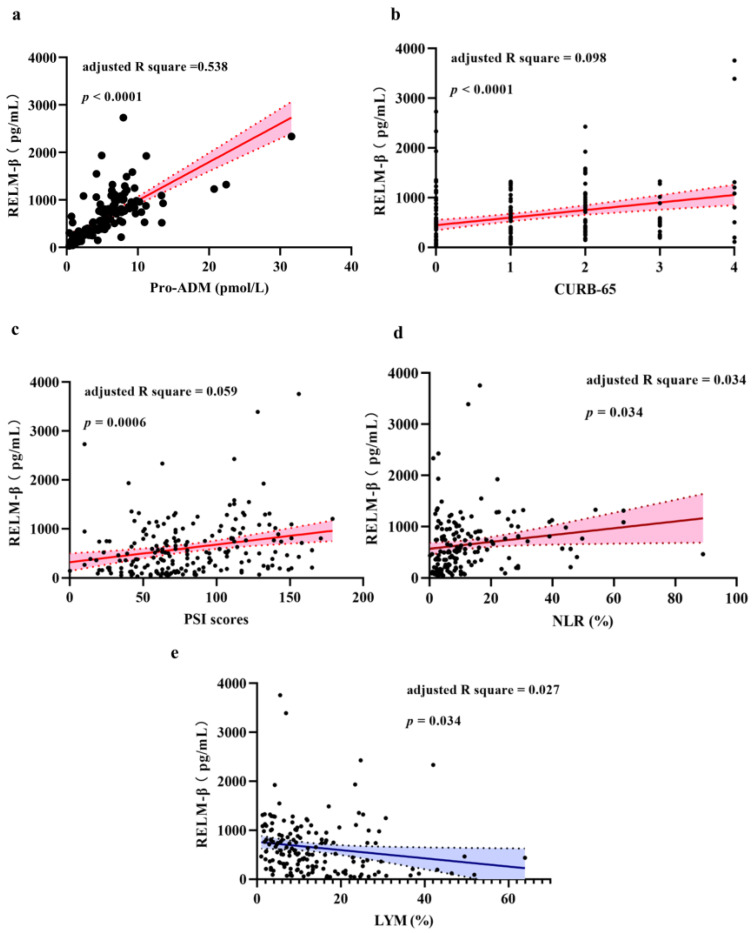
Correlation of resistin-like molecule beta (RELM-β) level with multiple clinical indicators across 226 patients with community-acquired pneumonia (CAP). Levels of RELM-β were significantly positively correlated with the level of proadrenomedullin (proADM) (**a**); confusion, urea, respiratory rate, blood pressure, and age ≥ 65 years old (CURB-65) score (**b**); pneumonia severity index (PSI) scores (**c**); and neutrophil-to-lymphocyte ratio (NLR) (**d**). RELM-β level was statistically negatively correlated with the lymphocyte percentage (LYM) (**e**).

We used the CURB-65 and PSI scoring systems to evaluate the severity of disease for all enrolled patients with CAP. In our sample of 226 patients, serum RELM-β levels were positively correlated with the level of proADM (adjusted R square = 0.538, *p* < 0.0001), CURB-65 score (adjusted R square = 0.098, *p* < 0.0001) and PSI score (adjusted R square = 0.059, *p* = 0.0006). In addition, the level of RELM-β at admission in patients with CAP was positively correlated with NLR (adjusted R-squared = 0.034, *p* = 0.034) and was negatively correlated with LYM (adjusted R-squared = 0.027, *p* = 0.034) (Figure 2 and Figure S1).

### 2.4. Value of RELM-β in Predicting Severity in CAP Patients

Receiver operating characteristic curves (ROCs) were used to assess the value of RELM-β and the clinical indicators for predicting SCAP (Table 2). The area under the curve (AUC) of the RELM-β of patients for predicting SCAP in patients with CAP was 0.794 (0.736–0.845) and the optimal cutoff value was 416.04 pg/mL. The AUCs of the CURB-65 and PSI scores of patients were 0.771 (0.706–0.828) and 0.791 (0.727–0.845), respectively. In addition, the AUC values of WBC, NEU%, LYM%, and NLR were 0.658 (0.587–0723), 0.735 (0.661–0.801), 0.756 (0.683–0.819), and 0.761 (0.688–0.824), respectively. However, although the ROC of RELM-β distinguishing patients with SCAP and patients with NSCAP was numerically higher than other indicators, there was no statistical difference (all *p* > 0.05) (Table S2). The multiple logistic regression analysis revealed that a combination of RELM-β and CURB-65 had an AUC value of 0.860 (0.803–0.906), with 84.55% sensitivity and 71.76% specificity when distinguishing SCAP patients from NSCAP, and its predictive ability was significantly superior to that of proADM or CURB-65 (both *p* < 0.05) (Table S2). The prognostic utility was substantially improved when combined RELM-β and PSI scores, instead of proADM alone, were applied to predictions in patients with CAP (*p* < 0.05) (Table 2 and Table S2).

### 2.5. Prognostic Ability of RELM-β in CAP Patients of 30-Day Mortality

Table 3 summarizes the ability of RELM-β and clinical indicators to predict the 30-day mortality of patients with CAP. The AUC for RELM-β was 0.777 (0.717–0.829). The optimal threshold for predicting 30-day mortality was 1006.14 pg/mL of RELM-β with a sensitivity of 57.89% and specificity of 87.44%. Predictions of 30-day mortality that combined CURB-65 or PSI with RELM-β were the most accurate (AUC of 0.844 (0.786–0.892) and 0.871 (0.816–0.915), respectively) (Table 3). Compared with proADM, when RELM-β and CURB-65 were combined to predict the 30-day mortality of patients with CAP, the prognostic utility significantly improved (*p* < 0.05). The clinical parameters of NEU% and CRP have no value in predicting the 30-day mortality of patients with CAP (both *p* > 0.05). 

**Figure 3:**
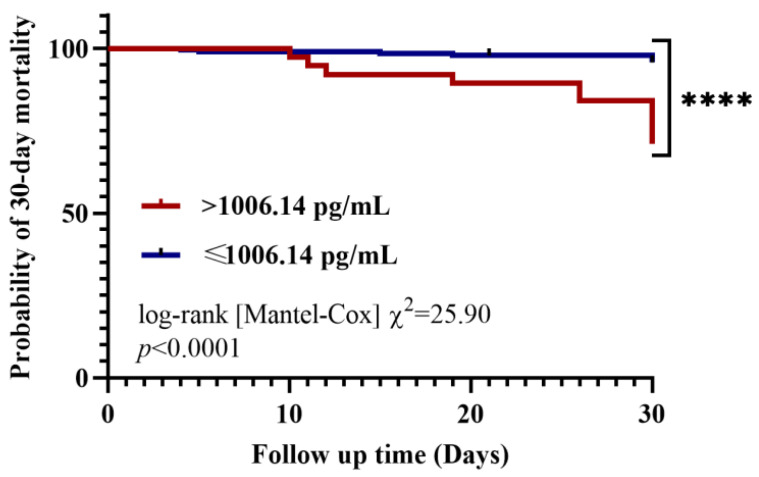
Kaplan–Meier analysis of 30-day mortality in patients with community-acquired pneumonia (CAP). Analysis was stratified by resistin-like molecule beta (RELM-β) levels. The RELM-β cutoff value (1006.14 pg/mL) is an optimal calculated cutoff value. **** *p* < 0.0001.

Kaplan–Meier survival curves were implemented to evaluate the relationship between the level of RELM-β and the 30-day mortality predictions for patients with CAP (Figure 3). Patients with CAP were divided into a higher RELM-β level group (serum concentration > 1006.14 pg/mL) and a lower RELM-β level group (serum concentration ≤ 1006.14 pg/mL) according to the optimal cutoff values calculated by ROC analysis. Statistically significant differences were observed in the mortality rate between the lower and higher RELM-β groups (log-rank (Mantel–Cox) χ^2^ = 25.90, *p* < 0.0001). The risk of death in patients in the higher-level RELM-β group was 7.40 (2.15–25.49) times greater than that of the lower-level group. The relationship between the remaining clinical indicators (including the level of proADM) and the 30-day mortality prediction of patients with CAP is described in detail in Figure S2.

The level of RELM-β, proADM, and clinical indicators were combined for multivariate Cox proportional hazards regression. Only heart rate and CRP were strong, independent predictors of 30-day survival (both *p* < 0.05) (Table S3). 

## 3. Discussion

The accurate diagnosis of CAP and early assessment of severity of disease are critical in the effective management, intervention, and precise treatment of patients [2]. Thus far, no research has examined the relationship between changes in serum RELM-β levels in patients and severity of illness. Our findings yield new insights into the prognostic risk factors for CAP. This study determined five key findings: (1) RELM-β levels in patients with CAP were statistically higher, particularly in non-survivors. (2) The level of RELM-β in the serum of patients with bacterial infection was statistically higher than that in patients with nonbacterial infection. However, the RELM-β level of the *Mp*^+^ group was significantly lower than that of the *Mp*^−^ group. (3) Increased levels of RELM-β displayed positive correlations with PSI and CURB-65. (4) Elevated levels of RELM-β were closely related to the severity of disease in patients with CAP. (5) RELM-β in the serum of patients with CAP was correlated with the death outcome within 30 days; the combination of clinical severity score and RELM-β can significantly improve mortality prediction ability. Increased levels of RELM-β in serum can assist clinicians in stratifying patients’ severity and prognostic risk at an early stage of admission.

RELM-β in humans is homologous to murine hypoxia-induced mitogenic factor [20], which is induced by hypoxia in the lungs, can significantly increase proliferation of microvascular smooth muscle cells, and can significantly contract pulmonary vasculature; therefore, RELM-β is a molecule of interest in studies on the mechanisms underlying CAP [21,22,23]. Previous studies have shown that RELM-β is associated with pathological processes that are related to lung diseases, such as airway fibrosis [24] and asthma [25]. In human pulmonary artery smooth muscle cells, chronic hypoxia can significantly up-regulate the level of RELM-β protein [26]. Additionally, LeMessurier et al. [27] demonstrated that the expression of RELM-β increases with the severity of asthma and acute bronchoconstriction. It has been reported that the concentration of RELM-β is high in the lung epithelial cells, fibroblasts, alveolar macrophages, and desquamated alveolar cells of patients with scleroderma-associated pulmonary hypertension and idiopathic pulmonary fibrosis, thus suggesting a profibrotic role in the lung [28]. Previous studies have identified that RELM-β is a cytokine associated with Th2 and is related to significant inflammatory and lung remodeling activity [16]. Similarly, we identified significantly elevated levels of RELM-β in the serum of patients with SCAP, particularly in non-survivors. This may be associated with organism hypoxia in patients with SCAP. 

Timely and reliable identifications of the underlying pathogens are critical for initiating effective and tailored antimicrobial treatment. We used a combination of multiple detection methods to determine the type of pathogen infection in CAP patients. Detection of respiratory pathogens in specimens taken directly from the lungs, such as by bronchoalveolar lavage fluid (BALF), pleural fluid sampling, or lung biopsy, is the “gold standard” for determining pneumonia etiology [29]. However, sputum has some advantages in determining pneumonia etiology, including its origin from the lower respiratory tract and noninvasive collection. In our study, bacteria were mainly detected through sputum culture unless the patient had undergone bronchoscopy testing or treatment. In those cases, the BALF was taken for loop-mediated isothermal amplification (LAMP) and bacterial culture. If at least one of the above methods detected positive bacteria, it was considered positive for specific bacteria. For *Mycoplasma pneumoniae*, polymerase chain reaction (PCR) detection using upper respiratory tract specimens and serology are not able to distinguish between infection and carriage [30]. Therefore, we mainly identify atypical pathogens (including *Mycoplasma pneumoniae*, *Chlamydia pneumoniae*, and *Legionella pneumophila*) through antibody detection using patient serum. Testing for influenza virus and other respiratory viruses in upper respiratory tract specimens using PCR was applied to evaluate patients with CAP in this study. Compared with other pathogen infections, our results indicate patients with bacterial pneumonia have a statistically significant increase in RELM-β. Similarly, Morampudi et al. [31] found that when mice suffer from colitis, the secretion of the goblet cell mediator RELM-β increases dramatically, and RELM-β promotes colitis by inducing changes in commensal bacteria via the induction of the antimicrobial lectin RegIIIb. Watanabe et al. [32] proved that RELM-β showed antimicrobial activity against *Staphylococcus aureus* and all methicillin-resistant *S. aureus* examined in a dose- and pH-dependent manner. Both RELM-β mRNA and protein expression were induced by heat-inactivated *S. aureus*, and RELM-β bound to the cell surface of *S. aureus*, followed by the destruction of the bacterial cytoplasm. However, its role in the development of CAP is unclear. Mycoplasma pneumoniae is a frequent cause of community-acquired respiratory infections in children and adults [33], but we found the RELM-β level of the *Mp*^−^ group was significantly higher than that of the *Mp*^+^ group. Regardless of whether the patient is infected with *Mp*, the concentration of RELM-β will also increase significantly as the patient’s disease gets worse. As there are no other studies on the RELM-β level of *Mycoplasma pneumonia* patients, the role of RELM-β in patients with *Mp* infection requires further study. We further stratified the patients with CAP according to their PSI and CURB-65 scores and discovered strong correlations between the RELM-β level and clinical scoring systems. These results suggest that RELM-β is a possible marker for assessing the severity of CAP, but the diagnostic value of RELM-β is reportedly poor. As an inflammatory regulator, RELM-β may be a potential biomarker of necrotizing enterocolitis (NEC), and the combination of RELM-β and thrombocytopenia was a reliable biomarker for the early diagnosis of NEC (AUC = 0.841), with 82.89% sensitivity and 93.21% specificity [34]. Our results show that the level of RELM-β was closely related to the severity of the disease in patients. However, in predicting patients with SCAP, the predictive ability of RELM-β was not significantly superior to other clinical indicators. The level of RELM-β can facilitate and objectively assess the severity of patients with CAP at the time of initial admission, particularly if the clinical severity score cannot be used normally owing to incomplete clinical data. Scarce data are obtained on the predicting prognostic accuracy of RELM-β in patients with CAP. Our results show that RELM-β performed well in predicting 30-day mortality, with an AUC value of 0.777, but its predictive ability was not significantly higher than NLR. NLR has been described to be correlated with the prognosis of several diseases [35,36]. Qiu et al. [37] demonstrated that NLR was independently related to the mortality of renal transplant recipients with SCAP and was an excellent biomarker with high prognostic performance for predicting poor prognosis (AUC = 0.88). In this study, while the ROC of RELM-β distinguishing patients with SCAP and patients with NSCAP was numerically higher than that of NLR, there was no statistical difference (*p* > 0.05). When RELM-β levels were combined with CURB-65 scores, the ability to predict mortality increased (AUC = 0.844, *p* < 0.05). The severity score alone is often insufficient to obtain satisfactory predictive accuracy. Our results show the addition of RELM-β to a clinical severity scoring method significantly improved their prognostic accuracy. ProADM is considered an excellent single predictor of short-term and long-term mortality [19]. In this study, the level of proADM was not better than that of RELM-β in predicting SCAP and 30-day mortality. When RELM-β was combined with CURB-65 or PSI, the ability of the new combinations to predict 30-day mortality was significantly higher than that of proADM.

Kaplan–Meier survival curves showed that CAP patients with serum RELM-β level < 1006.14 pg/mL on admission had a significantly longer survival time than patients with admission RELM-β ≥ 1006.14 pg/mL. The serum RELM-β level upon admission may be used as a prognostic factor to assist in judging the severity of the disease and can help in making informed decisions regarding treatment to increase the patient survival rate. In multivariate Cox proportional hazards regression, only heart rate and CRP were included in the final model (both *p* < 0.05). That is, in our study, the heart rate and CRP of patients with CAP were independent factors influencing the survival of patients within 30 days (Table S3). The levels of RELM-β and proADM in the serum of patients were excluded from the risk function equation. The level of RELM-β was related to the 30-day outcome of patients, but was not an independent factor, after eliminating confounding factors. 

This study has several limitations. Serum RELM-β levels were only measured upon admission, and linear dynamic investigations during the pathogenesis of CAP and follow-up changes in response to treatment should be further studied. Moreover, while our study enrolled several patients with comorbidities (Table 1), the pre-admission levels of RELM-β should be tested. Future studies should assess the value of RELM-β for predicting long-term mortality. 

The findings of this study demonstrate that the level of serum RELM-β measured upon admission is statistically elevated in patients with CAP with high severity scores, and is related to the etiology of CAP. Furthermore, the level of RELM-β was closely related to the disease severity and 30-day mortality of patients of CAP. Combining RELM-β levels with clinical scores significantly improved 30-day mortality predictions.

## 4. Materials and Methods 

### 4.1. Study Design and Population

This prospective, multicenter, observational study was conducted in 2017 on patients hospitalized in three hospitals, namely, Peking University People’s Hospital (PKUPH), Tianjin Medical University General Hospital, and Wuhan University People’s Hospital (ClinicalTrials.gov ID, NCT03093220). All patients with CAP were enrolled from the intensive care unit or the respiratory medicine department. Patients with a diagnosis of CAP were recruited for the study within 8 h of their admittance to the hospital. During this period, we recruited 15 age- and sex-matched healthy volunteers as HCs to provide normal RELM-β reference interval values. This study was employed according to the Declaration of Helsinki and was approved by the ethics committee of PKUPH (No. 2016PHB202–01). All patients signed an informed consent form prior to data collection. CAP and SCAP were determined according to the standard definition issued by the American Thoracic Society in 2007 [38]. SCAP was defined as the presence of at least one major criterion or at least three minor criteria. The major criteria were invasive mechanical ventilation and septic shock with the need for vasopressors; the minor criteria included respiratory rate ≥ 30 breaths/minute, oxygenation index ≤ 250, multipolar infiltrates, confusion or disorientation, uremia (blood urea nitrogen ≥ 20 mg/dL), leukopenia (WBC count < 4000 cells/mm^3^), thrombocytopenia (platelet count <100,000 cells/mm^3^), hypothermia (core temperature < 36 °C), and hypotension requiring aggressive fluid resuscitation. We recorded the following clinical characteristics within 8 h of admission: prior antibiotic treatment over 5 d, smoking status, underlying conditions, heart rate, T_max_, and respiratory rate, in addition to demographic information such as sex and age. Laboratory tests (WBC, LYM, NEU, NLR, CRP, and PCT) were performed within 24 h of admission, and imaging data were collected. The evaluation of SCAP and NSCAP, in addition to the evaluation of the PSI and CURB-65 scores of each patient with CAP, was conducted at the time of admission. Culture method was applied to identify bacterial pathogens of patients with CAP in clinical microbiology laboratories. Due to the high requirements for the cultivation of viruses, atypical pathogens and the long culture period [29], multiplex PCR based respiratory pathogen panels and LAMP were used to sensitively detect the viruses and atypical pathogens in sputum or alveolar lavage fluid of patients (Table S1). The primary endpoint of this study was the diagnosis of SCAP, and discharge from the hospital or hospital mortality was defined as a secondary endpoint. Structured telephone interviews were used to assess the outcome of CAP patients 30 days after admission (File S1: Questionnaire about the 30-day outcome of patients with community-acquired pneumonia).

### 4.2. Measuring RELM-β and proADM Levels in the Serum

Within 24 h of hospital admission, we collected peripheral venous blood in a sterile procoagulation tube and immediately centrifuged it to extract the serum. The serum of all participants was stored at −80 °C for further analysis. The level of RELM-β was analyzed in the serum samples twice by using quantitative enzyme-linked immunosorbent assay kits (Shanghai Enzyme-linked Biotech, Shanghai, China) according to the manufacturer’s instructions. The concentration of proADM was detected by the same method as above and subjected to subsequent analysis. The minimum concentration that can be detected by the kit is <0.1 pg/mL, and the coefficients of variation of inter-assay and intra-assay were <15% and <10%, respectively. The absorbance was detected using a 450 nm spectrophotometer (Multiskan FC, Thermo, Waltham, MA, USA), and the wavelength correction was set to 570 nm. The level of RELM-β was analyzed using the standard curve and ELISACalc 1.0 (Blue Gene, Shanghai, China).

### 4.3. Statistical Analyses

The distribution of continuous variable data was evaluated using the Kolmogorov–Smirnov test. The Kruskal–Wallis H test or the Mann–Whitney U test was used to analyze the continuous nonparametric data, which are described as the median (IQR). Categorical variables were presented as frequencies/percentages and were compared using the chi-square test or Fisher’s exact test. The continuous parametric data were expressed as mean ± SD, and post-hoc Tukey HSD test or Student’s *t*-test was used to assess the data. Categorical variables were presented as frequencies/percentages and were compared using Fisher’s exact test or the chi-square test. The area under the ROCs, cutoff value, sensitivity, and specificity were applied to assess the clinical performance of certain indicators. Spearman’s correlation coefficient was used to assess the linear relationship between clinical indicators and the level of RELM-β. Survival rates were compared using logarithmic rank tests, and the Kaplan–Meier survival analysis was used to create a 30-day survival curve. Cox proportional hazards regression analyses were used to analyze the effect of an array of variables on 30-day survival.

MedCalc Software version 15.2.0 (MedCalc Software, Ostend, Belgium), GraphPad Prism version 6.01 software (GraphPad Software, La Jolla, CA, USA), and SPSS statistics version 19.0 (IBM, Armonk, NY, USA) were utilized to perform statistical analyses and prepare figures. A two-tailed *p*-value less than 0.05 (typically ≤0.05) was considered statistically significant.

## Figures and Tables

**Table 1:** Demographic and clinical characteristics of the 226 subjects enrolled in this study

Characteristic	NSCAP (N = 112)	SCAP (N = 114)	*p*-Value
Male sex—no. (%)	80 (71.43)	82 (71.93)	0.525 ^†^
Age—years	55 (46.50–62.00)	57.72 ± 17.82	0.213 ^†††^
Smoking history—no. (%)	18 (16.07)	19 (16.67)	0.524 ^†^
Underlying diseases— no. (%)			
Chronic heart failure	5 (4.46)	10 (8.77)	
Diabetes mellitus	18 (16.07)	18 (15.79)	
Cerebrovascular disease	9 (8.04)	24 (21.05)	
Chronic liver disease	5 (4.46)	6 (5.26)	
Chronic renal disease	1 (0.86)	3 (2.63)	
Bronchiectasis	1 (0.86)	2 (1.75)	
Chronic obstructive pulmonary disease	1 (1.86)	9 (7.89)	
Antibiotic pre-treatment—no. (%)	36 (32.14)	37 (32.46)	0.776 ^†^
Physical examination			
T max (℃)	38.50 (37.88–39.20)	39.00 (38.00–39.83)	0.082 ^†††^
Respiratory frequency (times/min)	20 (18–22)	28 (22–32)	<0.0001 ^†††^
Heart rate	90 ± 14	102 ± 18	<0.0001 ^††^
Laboratory results			
WBC (×109/L)	7.53 (5.45–11.56)	11.20 (7.30–16.40)	<0.0001 ^†††^
NEU (%)	73.95 (65.93–82.33)	84.70 (78.40–90.70)	<0.0001 ^†††^
LYM (%)	16.55 (10.13–25.13)	8.00 (3.90–12.60)	<0.0001 ^†††^
NLR	4.18 (2.62–8.33)	10.54 (6.24–23.21)	<0.0001 ^†††^
CRP	51.49 (6.72–139.50)	105.00 (28.45–172.60)	0.018 ^†††^
PCT	0.11 (0.04–0.34)	0.61 (0.24–5.00)	<0.0001 ^†††^
ESR	33.00 (19.50–54.00)	55.00 (38.00–81.00)	0.0004 ^†††^
Chest X-ray			
Bilateral lung infection—no. (%)	29 (25.89)	96 (84.21)	<0.0001 ^†^
Pleural effusion—no. (%)	4 (3.57)	37 (32.46)	<0.0001 ^†^
Detected pathogen—no. (%)			
Bacteria ^a^	21 (18.75)	36 (31.58)	
Virus ^b^	10 (8.93)	21 (18.75)	
Atypical pathogen ^c^	11 (9.82)	1 (0.88)	
Mixed pathogen	7 (6.25)	7 (6.14)	
Unknown	63 (56.25)	49 (42.98)	
CURB-65			
Score points	0 (0–1)	2 (1–2)	<0.0001 ^†††^
PSI			
Score points	62.00 (42.00–72.00)	98.03 ± 37.61	<0.0001 ^††^
30-day mortality-no. (%)	0 (0.00)	19 (16.67)	<0.0001 ^†^

Descriptive statistics. Variables are expressed as numbers (percentages). Continuous variables are expressed as mean ± standard deviation (mean ± SD) when they met the normal distribution, and continuous nonparametric data are presented as median and interquartile ranges (25th and 75th percentiles). ^†^ Chi-square test or Fisher’s exact test; ^††^ Student’s *t*-test or analysis of variance with post-hoc Tukey HSD test; ^†††^ Mann–Whitney U or Kruskal–Wallis H test a: Including: *Pseudomonas aeruginosa*, *Stenotrophomonas maltophilia*, *Acinetobacter baumannii*, *Klebsiella pneumoniae*, *Staphylococcus aureus*, *Streptococcus pneumoniae*, *Escherichia coli*, *Haemophilus influenzae*, *Methicillin-resistant Staphylococcus aureus*, and *Pseudomonas aeruginosa* b: Including: *adenovirus*, *parainfluenza virus*, *rhinovirus*, *coronavirus*, *influenza virus*, *human metapneumovirus*, *respiratory syncytial virus*, *enzootic nasal tumor virus.* c: Including: *mycoplasma pneumonia*, *chlamydia pneumonia*, *legionella pneumophila*, and *mycobacterium tuberculosis complex.* Abbreviations: WBC: white blood cell; NEU%: neutrophil percentage; LYM%: lymphocyte percentage; CRP: C-reactive protein; PCT: procalcitonin; ESR: erythrocyte sedimentation rate; CURB-65: confusion, urea, respiratory rate, blood pressure, and age ≥65 years old; PSI: pneumonia severity index.

**Table 2:** Area under the curve (AUC) values and thresholds for predicting SCAP in CAP patients.

	AUC	95% CI	Sensitivity	Specificity	Threshold	*p*-Value
RELM-β	0.794	0.736–0.845	75.44	70.54	>416.04	<0.0001
WBC	0.658	0.587–0.723	36.94	89.77	>13.53	<0.0001
NEU %	0.735	0.661–0.801	70.75	66.67	>79.60	<0.0001
LYM %	0.756	0.683–0.819	67.62	75.00	≤10.3	<0.0001
NLR	0.761	0.688–0.824	59.62	83.33	>8.78	<0.0001
CRP	0.622	0.534–0.705	65.43	60.78	>66.72	0.0180
PCT	0.750	0.663–0.824	69.88	76.32	>0.31	<0.0001
CURB-65	0.771	0.706–0.828	77.27	65.88	>0	<0.0001
PSI	0.791	0.727–0.845	63.06	90.80	>86	<0.0001
proADM	0.720	0.641–0.791	91.30	58.23	>1.84	<0.0001
RELM-β + CURB-65	0.860	0.803–0.906	84.55	71.76	--	<0.0001
RELM-β + PSI	0.861	0.805–0.906	81.08	81.61	--	<0.0001

Abbreviations: CI: confidence interval; RELM-β: resistin-like molecules beta; WBC: white blood cell; NEU%: neutrophil percentage; LYM%: lymphocyte percentage; NLR: neutrophil-to-lymphocyte ratio; CRP: C-reactive protein; PCT: procalcitonin; CURB-65: confusion, urea, respiratory rate, blood pressure, and age ≥ 65 years old; PSI: pneumonia severity index; proADM: proadrenomedullin.

**Table 3:** AUC values and thresholds for predicting 30-day mortality in patients with CAP.

	AUC	95% CI	Sensitivity	Specificity	Threshold	*p*-Value
RELM-β	0.777	0.717–0.829	57.89	87.44	>1006.14	<0.0001
WBC	0.737	0.670–0.797	57.89	85.56	>15.21	0.0009
NEU %	0.606	0.527–0.681	86.67	49.01	>80.60	0.1552
LYM %	0.693	0.616–0.762	60.00	79.33	≤5.30	0.0190
NLR	0.698	0.621–0.767	60.00	81.21	>16.75	0.0170
CRP	0.515	0.426–0.602	41.67	80.00	≤12.80	0.8706
PCT	0.830	0.751–0.892	81.82	72.73	>1.33	<0.0001
CURB-65	0.764	0.698–0.822	73.68	71.02	>1	<0.0001
PSI	0.820	0.759–0.871	89.47	75.98	>106	<0.0001
proADM	0.723	0.644–0.794	92.86	52.99	>4.16	0.0001
RELM-β + CURB-65	0.844	0.786–0.892	89.47	71.02	--	<0.0001
RELM-β + PSI	0.871	0.816–0.915	89.47	73.18	--	<0.0001

Abbreviations: CI: confidence interval; RELM-β: resistin-like molecules beta; WBC: white blood cell; NEU%: neutrophil percentage; LYM%: lymphocyte percentage; NLR: neutrophil-to-lymphocyte ratio; CRP: C-reactive protein; PCT: procalcitonin; CURB-65: confusion, urea, respiratory rate, blood pressure, and age ≥ 65 years old; PSI: pneumonia severity index; proADM: proadrenomedullin.

## Supplementary Materials

The following are available online at https://www.mdpi.com/2076-0817/10/2/122/s1, Figure S1: Correlation of the level of resistin-like molecule beta (RELM-β) with procalcitonin (PCT) and C-reactive protein (CRP) across 226 patients with community-acquired pneumonia; Figure S2: Kaplan–Meier analysis of 30-day mortality in patients with community-acquired pneumonia (CAP). Analysis was stratified by proADM and other clinical indices; Table S1: Pathogen detection status of all CAP patients included in the study; Table S2: Pairwise comparison of ROC curves of RELM-β distinguishing patients with SCAP and patients with NSCAP; Table S3: Multivariate Cox regression analysis of patients with community-acquired pneumonia (stepwise method); File S1: Questionnaire about the 30-day outcome of patients with community-acquired pneumonia.
