# Supplementary material for: Predictive and Prognostic Utility of the Serum Level of Resistin-Like Molecule Beta for Risk Stratification in Patients with Community-Acquired Pneumonia
Source: Pathogens. 2021 Jan 25;10(2):122. doi: 10.3390/pathogens10020122 (PMC7912120; doi:10.3390/pathogens10020122)
Supplement: Supplementary file 1 [file pathogens-10-00122-s001.zip › pathogens-1041332/pathogens-1041332-supplementary/Figure S1.docx]

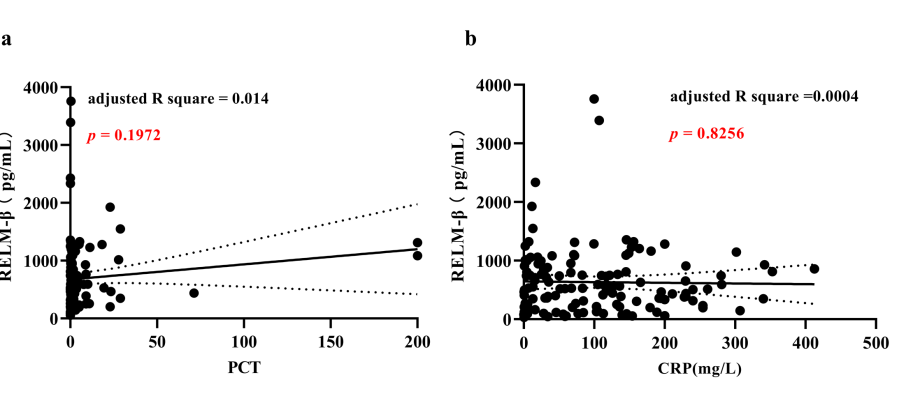


**Figure S1**. Correlation of resistin-like molecule beta (RELM-β) level with procalcitonin (PCT) (a) and C-reactive protein (CRP) (b) across 226 patients with community-acquired pneumonia
