# Supplementary material for: Predictive and Prognostic Utility of the Serum Level of Resistin-Like Molecule Beta for Risk Stratification in Patients with Community-Acquired Pneumonia
Source: Pathogens. 2021 Jan 25;10(2):122. doi: 10.3390/pathogens10020122 (PMC7912120; doi:10.3390/pathogens10020122)
Supplement: Supplementary file 1 [file pathogens-10-00122-s001.zip › pathogens-1041332/pathogens-1041332-supplementary/Figure S2.docx]

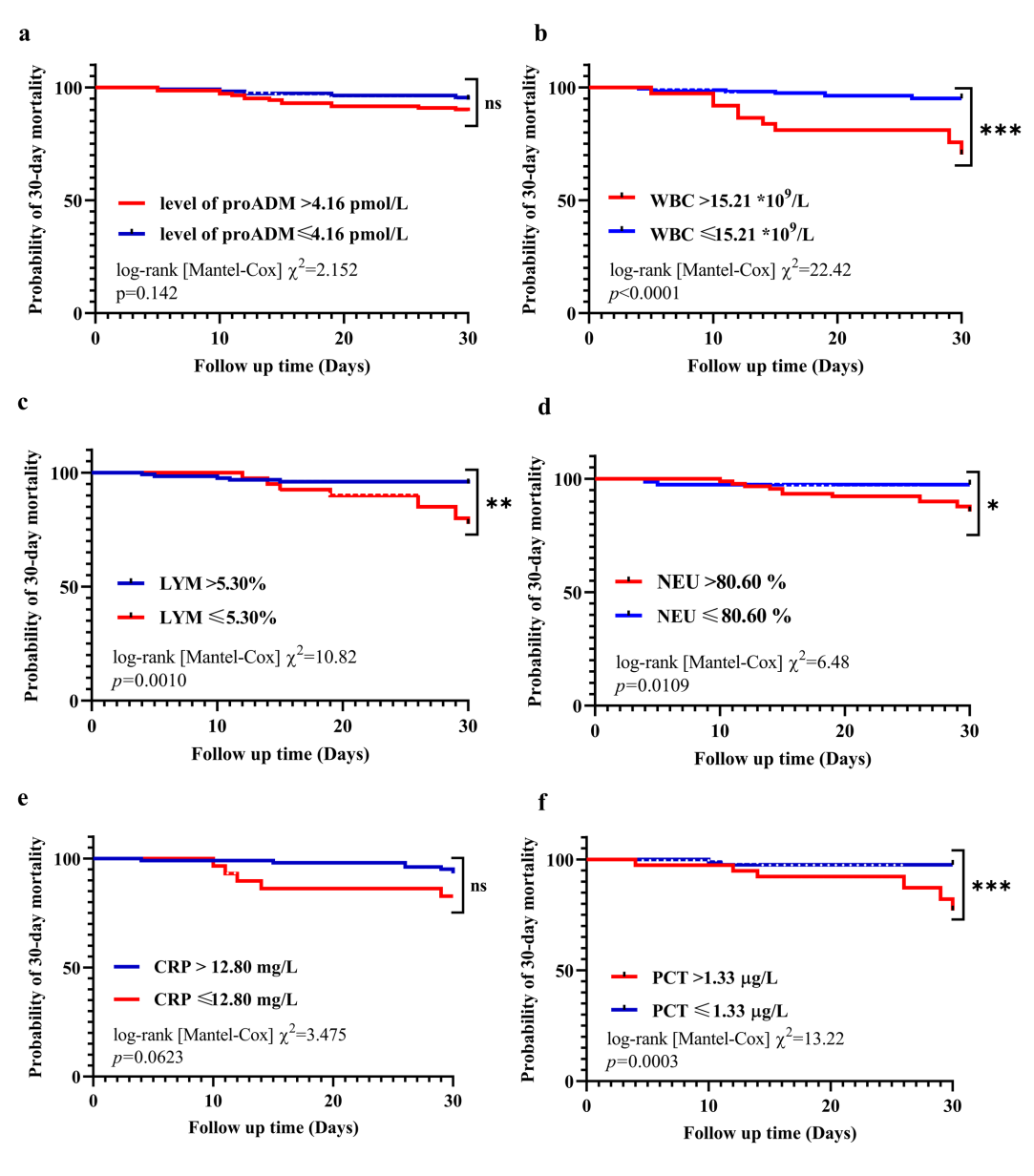

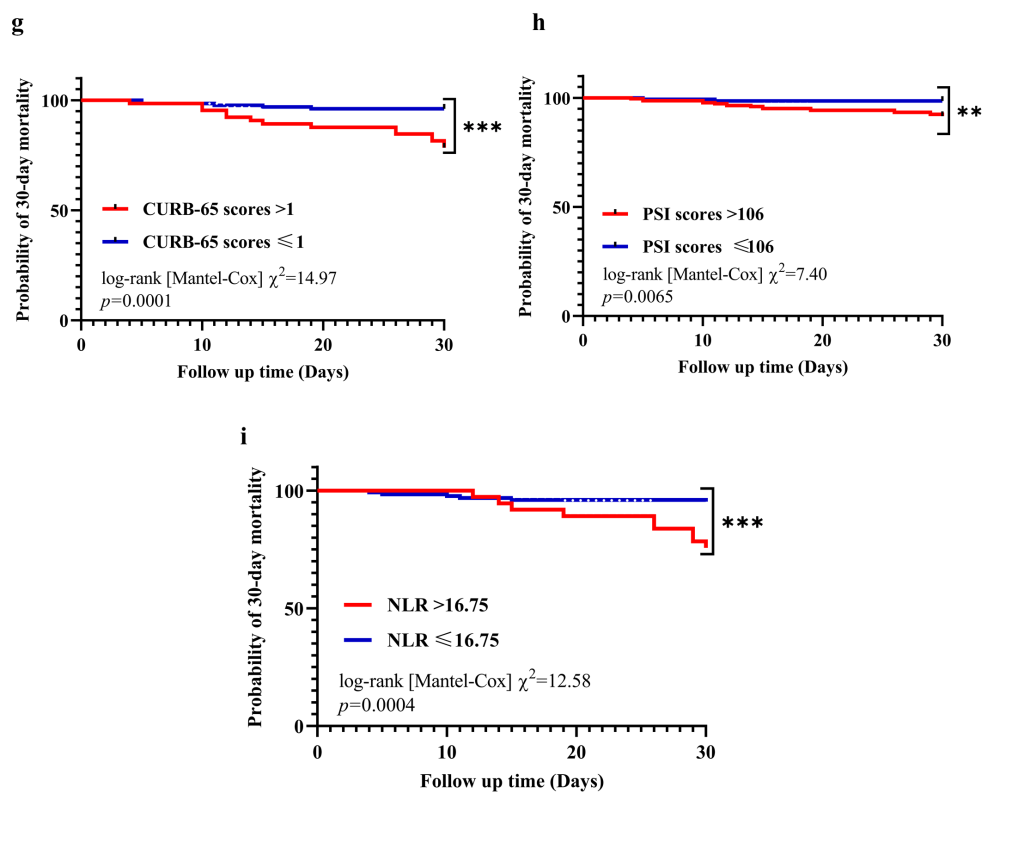


**Figure S2**. Kaplan–Meier analysis of 30-day mortality in patients with community-acquired pneumonia (CAP). Analysis was stratified by proADM (a) and other clinical index. The cutoff values are optimal calculated cutoff value. WBC (b) : white blood cell; NEU (d): neutrophil percentage; LYM (c) : lymphocyte percentage; NLR (i): neutrophil-to-lymphocyte ratio; CRP (e): C-reactive protein; PCT (f) : procalcitonin; CURB-65 (g): confusion, urea, respiratory rate, blood pressure, and age ≥ 65 years old; PSI (h): pneumonia severity index; proADM: proadrenomedullin. * *p* < 0.05, ** *p* < 0.01, *** *p* < 0.001, **** *p* < 0.0001
