# Supplementary material for: Predictive and Prognostic Utility of the Serum Level of Resistin-Like Molecule Beta for Risk Stratification in Patients with Community-Acquired Pneumonia
Source: Pathogens. 2021 Jan 25;10(2):122. doi: 10.3390/pathogens10020122 (PMC7912120; doi:10.3390/pathogens10020122)
Supplement: Supplementary file 1 [file pathogens-10-00122-s001.zip › pathogens-1041332/pathogens-1041332-supplementary/Table S2.docx]

**Table S2.** Pairwise comparison of ROC curves of RELM-β distinguishing patients with SCAP and patients with NSCAP

|  | **Difference**  **between areas** | **Standard Error^a^** | **95% CI** | **z statistic** | **p value** |
| --- | --- | --- | --- | --- | --- |
| RELM-β vs. proADM | 0.097 | 0.063 | -0.026 to 0.220 | 1.543 | 0.123 |
| RELM-β vs. WBC | 0.096 | 0.105 | -0.110 to 0.302 | 0.911 | 0.362 |
| RELM-β vs. NEU | 0.148 | 0.082 | -0.013 to 0.309 | 1.802 | 0.072 |
| RELM-β vs. LYM | 0.098 | 0.076 | -0.050 to 0.246 | 1.297 | 0.195 |
| RELM-β vs. NLR | 0.100 | 0.079 | -0.054 to 0.254 | 1.273 | 0.203 |
| RELM-β vs.CURB-65 | 0.011 | 0.103 | -0.191 to 0.214 | 0.110 | 0.913 |
| RELM-β vs. PSI | 0.051 | 0.131 | -0.206 to 0.309 | 0.389 | 0.075 |
| RELM-β vs. PCT | 0.006 | 0.141 | -0.270 to 0.283 | 0.046 | 0.963 |
| RELM-β vs. ESR | 0.162 | 0.127 | -0.087 to 0.412 | 1.275 | 0.202 |
| RELM-β vs. CRP | 0.162 | 0.144 | -0.121 to 0.445 | 1.124 | 0.261 |
| RELM-β+CURB-65 vs. proADM | 0.096 | 0.041 | 0.015 to 0.177 | 2.318 | 0.021 |
| RELM-β+CURB-65 vs. CURB-65 | 0.081 | 0.034 | 0.015 to 0.147 | 2.399 | 0.016 |
| RELM-β+PSI vs. proADM | 0.103 | 0.041 | 0.022 to 0.184 | 2.501 | 0.012 |

 a DeLong test

Abbreviations: CI: confidence interval; RELM-β: resistin-like molecules beta; WBC: white blood cell; NEU: neutrophil percentage; LYM: lymphocyte percentage; NLR: neutrophil-to-lymphocyte ratio; CRP: C-reactive protein; PCT: procalcitonin; ESR: erythrocyte sedimentation rate; CURB-65: confusion, urea, respiratory rate, blood pressure, and age ≥ 65 years old; PSI: pneumonia severity index; proADM: proadrenomedullin.
