# Supplementary material for: Predictive and Prognostic Utility of the Serum Level of Resistin-Like Molecule Beta for Risk Stratification in Patients with Community-Acquired Pneumonia
Source: Pathogens. 2021 Jan 25;10(2):122. doi: 10.3390/pathogens10020122 (PMC7912120; doi:10.3390/pathogens10020122)
Supplement: Supplementary file 1 [file pathogens-10-00122-s001.zip › pathogens-1041332/pathogens-1041332-supplementary/Table S3.docx]

**Table S3**. Multivariate Cox regression analysis of patients with community-acquired pneumonia (stepwise method).

| **Variable** | **B** | **SE** | **Wald χ^2^** | ***p* value** | **RR (95%CI)** |
| --- | --- | --- | --- | --- | --- |
|  |  |  |  |  |  |
| Heart rate | 0.146 | 0.060 | 5.987 | 0.014 | 1.157 (1.029-1.300) |
| CRP | −0.033 | 0.016 | 4.370 | 0.037 | 0.967 (0.938–0.998) |

CRP: C-reactive protein
